# Development and psychometric properties of the methamphetamine decisional balance scale (METH-DBS) for young adults

**DOI:** 10.1186/s13011-018-0175-0

**Published:** 2018-10-29

**Authors:** Maryam Khazaee-Pool, Leila Jahangiry, Tahereh Pashaei, Farhad Ramezani-badr, Haidar Nadrian, Koen Ponnet

**Affiliations:** 1 Department of Health Education and Promotion, School of Public Health, Zanjan University of Medical Sciences, Zanjan, Iran; 2 Department of Health Education and Health Promotion, Health Faculty, Tabriz University of Medical Sciences, Tabriz, Iran; 3 Environmental Health Research Center, Research Institute for Health Development, Kurdistan University of Medical Sciences, Sanandaj, Iran; 4 Department of Public Health, Faculty of Health, Kurdistan University of Medical Sciences, Sanandaj, Iran; 5 Department of Critical Care Nursing, School of Nursing and Midwifery, Zanjan University of Medical Sciences, Zanjan, Iran; 6 Department of Communication Sciences, Faculty of Political and Social Sciences, imec-mict-Ghent University, Ghent, Belgium

**Keywords:** Methamphetamine, Decisional balance, Scale development, Psychometrics, Young adults

## Abstract

**Background:**

Drug misuse is a major problem that has an extreme negative effect on people’s health. Methamphetamine (MA) is frequently used by young adults, despite its harmful consequences. The Transtheoretical Model (TTM) has been known to be very effective in explaining both the achievement and cessation of several health-related behaviors. Therefore, in this study, the TTM was used toward the domain of immoderate MA use among young adults. This study aimed to test the validity and reliability of a decisional balance scale for MA use in young adults.

**Methods:**

A multi-phase scale development approach was used to develop the scale. First, 41 university students enrolled in a qualitative study that generated content for a primary set of a 40-item instrument. In order to produce a pre-final version of the instrument, face and content validity were calculated in the next step. The instrument validation was assessed with a sample of 250 university students. Then, the construct validity (exploratory and confirmatory), convergent validity, discriminate validity, internal consistency applying test-retest reliability, and Cronbach’s alpha of the scale were measured.

**Results:**

Forty items were initially generated from the qualitative data. After content validity, this amount was reduced to 25 items. The exploratory factor analysis revealed four factors (self and other cons, coping and social pros) containing 21 items that jointly accounted for 55.24% of the observed variance. The confirmatory factor analysis indicated a model with appropriate fitness for the data. Cronbach’s alpha coefficient for the dimensions ranged from .74 to .87, and the Intraclass Correlation Coefficient (ICC) ranged from .83 to .91, which is within acceptable ranges.

**Conclusion:**

The findings showed that the Methamphetamine Decisional Balance Scale is a valid and reliable scale that increases our ability to study motivational factors related to MA use among young adult. Consequently, the instrument could be applied in both practice and future studies.

## Background

Based on a report from the World Health Organization, drug addiction is a chronic and frequent disorder that threatens public health and society’s well-being worldwide [1]. Methamphetamine (MA) is a highly addictive stimulant and one of the most commonly used illicit drugs in the world [2]. Particularly, MA use by young adults is a serious social concern. National data in the United States show that the prevalence of MA use among individuals aged 12 or older increased from 353,000 in 2010 to 569,000 in 2014 [3] in spite of its being illegal.

MA abuse leads to serious adverse health consequences, including widespread sleep deficiency, phobias, and poor health [4]. Moreover, in general, efficacious medications for treating MA misuse are not available [5–7]. Although any level of MA use for youths is illegal, extreme consumption results in an increased risk of serious negative sequelae such as accidents, unprotected sex, and interpersonal violence [8, 9].

According to the Iranian Drug Control Headquarters (DCHQ), 5.39% of people aged 15 to 64 in Iran are drug users [10]. Furthermore, there is evidence of a growing rate of MA use in Iran [11]. The most mentioned reasons for the growth of MA use in Iran are its easy accessibility, curiosity about having new experiences, low price compared to other drugs, lack of awareness of its complications, and expectations of improved sexual performance [12]. Consequently, some young adults believe that MA use has more benefits than side effects. A better understanding of the benefits and side effects of consuming MA may be helpful for programs that aim to prevent substance abuse among adolescents [13–15]. Considering the high prevalence of MA dependence, supporting a person’s motivation to change is the main aim of prevention programs. A key construct associated with someone’s motivation to change is decisional balance (DB), which was developed based on the conflict theory of decisions by Janis and Mann [16]. Both authors assumed that decision making about a specific behavior is based on the person’s consideration of possible gains and losses. The pros and cons of DB are generally used in the field of addiction treatment [17]. Based on the Janis and Mann framework, there are four content structures of pros and cons that are usually considered in this process: (a) losses/gains for oneself, (b) losses/gains for significant others, (c) self-approval or -disapproval, and (d) approval or disapproval from significant others. DB is regularly categorized into two groups of pros and cons, which may be anchored to either keeping or varying a target behavior.

DB has also been linked to the transtheoretical model (TTM), which refers to changes in behavior and its causal motivation [17]. The TTM is an integrative model that measures a person’s readiness to adopt different, healthier behaviors and prepares approaches to guide the individual in making these changes. The TTM considers behavior modification to be a process that develops over time, including progress via five stages of change: pre-contemplation, contemplation, preparation, action, and maintenance. Pros and cons not only make evidence available about the positive and negative beliefs toward a behavior but may also function as indicators for preparation to change [18]. Certainly, persons who progress further through the stages of change desire to report more pros and fewer cons of change as well as more cons and fewer pros of risky behavior. These trends appear with a range of risky behaviors [19], such as cigarette smoking among young adults [20]. However, there is no formal DB instrument specific to MA at this time.

Pros and cons are constructs similar to those in other cognitive-motivational dimensions such as expectancies and motives. Although DB instruments for tobacco use have been developed [21, 22], instruments for assessing motives for MA use do not exist. Furthermore, although motives may equate to the pros of DB, motivational evaluations do not address the perceived costs or adverse aspects of MA use, which is a key element that has been identified to be predictive of behavior change [22]. On the other hand, the outcome expectancies for MA consumption may also be associated with pros and cons [23]. Expectancies display overall cognitions about the probable consequences of intake behavior, while pros and cons report motivational elements specific to a person’s decisions about future behavior.

Given the existing theoretical dissimilarities, the development of a DB instrument for MA may be ideally suited to identify patterns of MA use. Pros refer to the benefit of doing a behavior and can increase the interest and intention of doing a behavior, while cons refer to the barriers to doing behaviors and the negative effects involved with doing those behaviors. Cons can decrease one’s interest in doing a behavior like MA. In other words, the pros and cons for using MA will refer to what increases or decreases the interest in using MA, thus providing information about what features of MA are observed as interesting versus aversive in young adults. For example, young adults may consider greater relaxation due to MA use as an initiator, or pro, and character changes as a disincentive, or con. However, no validated instrument to measure the pros and cons has yet been developed regarding MA use. Therefore, the aim of the present study was to develop and test the psychometric properties of a newly developed scale called the METH-DBS. The scale can be used to explore the pros and cons influencing Iranian young adults’ MA use and potentially reveal areas to apply interventions to prevent MA use among young adults. To establish the validity of METH-DBS, the relationship between the scale scores will be associated with the scores of four potentially associated constructs, namely, self-cons, coping pros, social pros, and other cons.

## Methods

### Research design

The present study was approved by the Ethics Committee of Zanjan University of Medical Sciences, and informed written consent was obtained from all participants. All participants were students of the Zanjan University of Medical Sciences in Zanjan, Iran. We performed this study in two phases. In the first phase, items were generated to develop the scale (in Farsi). A qualitative study was performed to create the primary suggestions for potential items regarding the perceived pros and cons of MA use. After determining the most suitable phrasing for each item, face and content validity were assessed.

In the second phase, the items were administered from a new sample. First, an exploratory factor analysis (EFA) was performed to find the principal factor structure, and items with insufficient loadings were removed. Then, confirmatory factor analysis (CFA) of the measurement model was applied in order to assess the coherence between the data and the structure. Thereafter, the final instrument was administered from an independent sample to check its factor structure. Furthermore, the convergent and discriminant validity and the internal consistency of the new scale were calculated. Afterward, test-retest reliability was assessed by means of an independent sample of 30 students.

### Phase 1: Item generation and instrument development phase

A qualitative study was conducted to develop a scale for assessing the pros and cons of MA use in young adults. Three focus group discussions (FGDs) among 24 young adults (8 participants per focus group) and 17 semi-structured interviews were conducted with a focus on the pros and cons of MA consumption based on DB constructs of the TTM. The students were recruited from October through December 2016.

A two-stage strategy was used to reach the participants. In the first stage, a total of 24 young adults (*M* age = 23.92, *SD* = 2.62, 13 males) were recruited from the schools of pharmacy, health, dentistry, nursing, and medicine that are affiliated with the Zanjan University of Medical Sciences. Attempts were made to reach students with varied demographic background characteristics. The researchers recruited participants via email. Respondents could sign up for the interviews by sending a message to the researchers. Written informed consent was obtained, and the participants were informed that the provided information would be kept private and confidential.

During the FGDs, the participants first completed questions on (a) sociodemographic (age, gender, year in university, occupational status, educational level, marital status, fields of study) and (b) their non-prescription use of legal and illegal drugs both in the last months and over the course of their lives. Then, students were questioned about the pros and cons of MA consumption related to their own experiences or, in case they had no experience, their observations of others’ experiences. Finally, based on Janis and Mann’s (1977) framework, they were encouraged to discuss personal losses/gains from MA consumption, others’ losses/gains, self-approval and -disapproval, and approval and disapproval from others [16]. The sessions were facilitated by defining addiction and using a semi-structured interview that started with an open-ended question: “What are the pros and cons of MA use for young adults?” Then, based on the participants’ answers, questions were generated to have a discussion. Analytic ideas were recorded by memo writing. The FGDs also allowed the researchers to find potential respondents for the individual interviews.

In a second stage, students from the same schools were recruited to participate in semi-structured interviews, with a maximum duration of 90 min. Among the 24 participants from the FGDs, seven mentioned during the FGDs that they had used MA. These participants were requested to supply additional data through individual interviews. Then, 10 additional students were recruited from different age groups, from different socioeconomic backgrounds, and with varying educational levels. The recruiters tried to reach participants living in different situations at the time of the interviews, with most participants living in dormitories and the others living with their families. Among the 10 students, three mentioned MA use. Of all MA users, their first experience with MA started at about age 21 (*SD* = 1.73, range: 19–24), and they described using MA for an average of 5 days (*SD* = 7.84) in the last 10 months. During the interviews, participants were questioned about their personal experiences of the pros and cons and also questioned using organized prompts concerning the possible factors of pros and cons (e.g., health, social, academic). This information was used to construct the phrasing of the items.

#### Data analysis

Inductive thematic qualitative content analysis (a bottom-up method to explore the data) was employed based on the Braun and Clarke method in order to converge and compare themes among participant data. Themes were clustered according to participants’ views and experiences about the cons and pros of MA. Using this method, we recognize themes based on the primary codes and categories. As such, the units of analysis are the entire interviews [24]. Data analysis started during the data gathering. Each FGD and individual interview was transcribed literally and analyzed before the next FGD or interview was accomplished. Thorough understanding of the data was reached by frequently reading the transcriptions.

The lists of pros and cons were generated with rate counts (available from the first author upon request). Answers that occurred many times or that converged on a theme were maintained as items. Answers that were unclear (e.g., “I had pleasure using it”), vague (e.g., “I think it’s worth for your senses”), or not eligible as cons/pros (e.g., “There are no dues on it”) were excluded. Finally, items that were only mentioned once were also omitted.

As mentioned above, the first draft of the scale was developed on the basis of the findings of the FGDs and the individual interviews. The pre-final draft of the Methamphetamine Decisional Balance Scale (METH-DBS) for young adults contained 40 items (in Farsi) that could be answered on a 5-point response scale (1 = *not at all important*; 5 = *very important*) for maximizing variability within the answers and measuring the importance of each statement for participants’ decision to use MA. All items were reread and revised for simplicity and wordiness. Items were phrased as statements about the positive and negative features of MA use. Then, content and face validity were tested to develop the pre-final version of the scale.

##### Content validity

We applied qualitative and quantitative content validity for METH-DBS. An expert panel that consisted of a group of researchers who specialized in drug addiction and psychometrics evaluated the content validity of the METH-DBS. In the qualitative phase, the instrument was assessed in terms of wording, item allocation, grammar, and scaling. Furthermore, the content validity index (CVI) and the content validity ratio (CVR) were tested in the quantitative phase. Clarity, simplicity, and relevance of the items were measured by a CVI assessment [25, 26]. A Likert-type ordinal scale with four possible responses was used for measuring the CVI. The responses were rated from 1 = *not relevant, not simple, and not clear* to 4 = *very relevant, very simple, and very clear*. The CVI was measured as the proportion of items that acquired a rating of 3 or 4 by the experts [27]. A CVI score of more than .80 for *each item* was acceptable [28]. Furthermore, the essentiality of each item was measured by the CVR. Each item was scored by experts as 1 = *essential*, 2 = *useful but not essential*, or 3 = *not essential* for measuring the CVR [27]. According to the Lawshe Table [29]*,* each item with a CVR > .62 was considered to be satisfactory and was kept.

In the quantitative phase, items with a CVR more than .62 and a CVI more than .80 were accepted. Overall, 11 items were removed, resulting in a 29-item pool. Furthermore, the experts reviewed the scale regarding wording, grammar, and item allocation. The 29-item pool remained in the analyses below and contained either a positive belief (pro) or a negative belief (con) that might occur to an individual who is deciding whether to use MA or to start. Each item could be answered on a 5-point scale: 1 = *not important at all*, 2 = *slightly important*, 3 = *moderately important*, 4 = *very important*, 5 = *extremely important.*

##### Face validity

Both qualitative and quantitative methods were used to measure the face validity of the METH-DBS. In the qualitative phase, 10 young adults were asked to assess each item of the METH-DBS and to indicate if they felt it was difficult or ambiguous to answer the items. Based on the participants’ viewpoints, the vague items were revised. In the quantitative stage, the impact score (frequency × importance) was measured to indicate the percentage of students who recognized items as important or quite important on a 5-point Likert scale. Consistent with other studies [25, 30, 31], items were found to be suitable if they had an impact score equal to or greater than 1.5 (which equates to a frequency of 50% or greater and a mean importance of 3 on the 5-point Likert scale) [32]. Overall, four items had an impact score equal to or lower than 1.5, and 25 items had an impact score ranging from 1.8 to 5. As such, the first form of the instrument contained 25 items.

### Phase 2: Psychometric evaluation of the methamphetamine decisional balance scale (METH-DBS) for young adults

In order to test the psychometric properties of the METH-DBS in a wider setting, a cross-sectional study was carried out in Zanjan, Iran, from February 2017 to April 2017. The participants were university students from the schools of pharmacy, health, dentistry, nursing, and medicine at Zanjan University of Medical Sciences. A multistage random sampling method was used based on sex ratio, educational levels, and field of study. Eight hundred students who were at least 19 years old were contacted based on phone numbers and emails provided by the Education Department. Participants were contacted irrespective of their MA use experiences. After the study objectives were explained via telephone and email, 250 students agreed to participate. Table 1 provides the participants’ descriptive characteristics.

**Table 1:** Characteristics of the study sample

	EFA sample (*n* = 250)	CFA sample (*n* = 189)	Test-retest sample (*n* = 30)
	Number (%)	Number (%)	Number (%)
Age (years)			
19–24	103 (41.2)	105 (55.56)	8 (26.67)
25–29	126 (50.4)	68 (35.98)	17 (56.67)
30 and above	31 (12.4)	16 (8.46)	5 (16.66)
Mean (SD)	23.62 (2.74)	24.46 (3.1)	26.12 (3.24)
Range	19–32	20–32	23–32
Gender			
Female	127 (50.8)	127 (67.2)	11 (36.66)
Male	123 (49.2)	62 (32.8)	19 (63.33)
Occupational status			
Unemployed	215 (86)	165 (87.3)	21 (70)
Employed	35 (14)	24 (12.7)	9 (30)
Educational Level			
Bachelor	47 (18.8)	32 (16.93)	8 (26.7)
Master degree	54 (21.6)	36 (19.05)	7 (23.3)
Doctorate	149 (59.6)	121 (64.02)	15 (50)
Marital status			
Single/divorced/widowed	171 (68.4)	147 (77.8)	21 (70)
Married	79 (31.6)	42 (22.2)	9 (30)
Fields of study			
Health	43 (17.2)	43 (22.75)	6 (20)
Dentistry	58 (23.2)	31 (16.4)	5 (16.7)
Nursing	44 (17.6)	33 (17.46)	7 (23.3)
Medicine	61 (24.4)	39 (20.63)	6 (20)
Pharmacy	44 (17.6)	43 (22.76)	6 (20)
Having experience of MA use			
Yes	9 (3.6)	5 (2.64)	1 (3.3)
No	241 (96.4)	184 (97.36)	29 (96.7)
Age of first experience of MA			
19–24	5 (55.56)	3 (60)	1 (100)
25–29	3 (33.33)	1 (20)	0
30 and above	1 (11.11)	1 (20)	0

After the main investigator had a short interview with each participant and provided information about the aim of the study, students who agreed to participate completed the METH-DBS. Besides the METH-DBS, demographic questions about the participants were included regarding age, level of education, fields of study, occupational status, marital status, and gender. Furthermore, respondents were questioned as to whether they had experiences of MA use as well as how old they were at their first experience. Trained investigators performed face-to-face interviews for data collection.

### Measures

Due to the lack of suitable Iranian validated questionnaires about MA use, the Decisional Balance Inventory (DBI) Adolescent Form for Smoking was used to establish the validity of the METH-DBS [33].

The DBI is a self-report scale that emphasizes either a positive thought (pro) or a negative thought (con) that might occur to an individual who is deciding whether to smoke. The original DBI developed by Velicer (1998) included 24 items that measured the opinions of adolescents about the harms and benefits of smoking [22]. The brief DBI developed by Pallonen (1998) consists of 12 items [33]. The shortened DBI measures one of the main constructs of the TTM and contains three dimensions, i.e., cons of smoking (six items), social pros (three items), and coping pros (three items), and each item ranged on a 5-point Likert-type scale (1 = *not important* to 5 = *extremely important*). So, the minimum score is 12 and the maximum is 60. The DBI has proven to have good validity and reliability in the Iranian population [30]. In the present study, Cronbach’s alpha was .82, indicating acceptable reliability.

#### Statistical analysis

Several statistical methods were performed to evaluate the psychometric properties of the METH-DBS. They are presented as follows.

### Validity

#### Construct validity

After the item analysis, the 25 remaining items were used to assess construct validity using both EFA and CFA. Additionally, both convergent validity and divergent validity were assessed.

##### Exploratory factor analysis

EFA was applied to identify the main factors of the METH-DBS. According to the number of items in the METH-DBS, which was multiplied by 5–10 as suggested by [34, 35], the sample size was assessed. The preferred maximum required sample size was thus determined to be 250 young adults. These participants were recruited from the different schools of Zanjan University of Medical Sciences, including pharmacy, health, dentistry, nursing, and medicine (see data collection section). A principal component analysis (PCA) with varimax rotation was applied to extract the main factors. The Kaiser-Meyer-Olkin (KMO) test and Bartlett’s test of sphericity were applied to evaluate the adequacy of the sample for the factor analysis [36]. Any factor with an eigenvalue of more than 1 was considered acceptable for factor extraction, and a scree plot was performed to specify the number of factors. Factor loadings equal to or greater than .40 were considered acceptable [37].

##### Confirmatory factor analysis

A CFA was applied to assess the coherence between the data and the structure. Considering possible attrition related to test-retest analysis, we planned to recruit a separate sample of 189 university students from the schools of pharmacy, health, dentistry, nursing, and medicine affiliated with the Zanjan University of Medical Sciences. Assigning 5–10 persons to each item, a sample size of 189 (9 × 21 = 189) was estimated [25, 35]. The model fit was evaluated using multiple fit indices. As recommended, various fit indices measuring relative chi-square, root mean square error of approximation (RMSEA), goodness of fit index (GFI), comparative fit index (CFI), normed fit index (NFI), non-normed fit index (NNFI), and standardized root mean square residual (SRMR) were taken into account [38]. The GFI, CFI, NFI, and NNFI ranged between 0 and 1 [39], but values of .90 or above are commonly considered satisfactory model fits [38]. An RMSEA value range from .08 to .10 indicates an average fit. Values below .05 represent a good fit for SRMR, but values between .05 and .08 and between .08 and .10 indicate a close fit or are satisfactory, respectively [40].

##### Convergent and divergent validity

To assess convergent and divergent validity, the 189 university students completed the Iranian validated version of the DBI [30]. Initially, we measured item-convergent validity by examining the correlations between the item scores and the subscale scores of the METH-DBS using the Spearman correlation coefficient. We expected that, for each subscale of the METH-DBS, the item scores of the subscale (e.g., con) would correlate more with the total score of the respective subscale (e.g., con) rather than the total score of the subscales (e.g., pro). Correlation values ranging from 0 to .20 are considered poor; from .21 to .40, fair; from .41 to .60, good; from 0.61 to 0.80, very good; and more than .81, excellent [41]. Item-convergent validity is achieved when each item has considerably greater correlation with its own instrument compared with the other instruments, and item divergent validity is achieved when each item has less correlation with other instruments [42]. Then we evaluated the convergent and divergent validity of four subscales of the METH-DBS (self-cons, other cons, coping pros, and social pros) compared to the abovementioned validated inventory (DBI) [30]. Convergent validity is confirmed when a subscale of the METH-DBS correlates moderately with the DBI (correlation .21 or more). We expected moderate correlations between the pro subscale of the METH-DBS and the pro subscale of the DBI as well as between the con subscale of the METH-DBS and the con subscale of the DBI. A poor correlation was found between a subscale of the METH-DBS and the DBI.

### Reliability

#### Internal consistency

Cronbach’s alpha coefficient was used to measure the internal consistency of each item, the whole scale, and each subscale (self cons, other cons, coping pros, and social pros) of the METH-DBS. Alpha values of .70 or above were considered acceptable [43].

#### Test-retest reliability

Test-retest reliability was used to test the METH-DBS’s stability by assessing the intraclass correlation coefficient (ICC). Fifty university students were emailed and asked to participate, of which 30 students agreed to participate. The METH-DBS was re-administered to these students 2 weeks after the first completion. ICC values of .40 or above are considered acceptable [44]. All statistical analyses, except CFA, were applied using SPSS 22.0 [45]. The CFA was performed using AMOS version 22 of SPSS [46].

## Results

A total of 250 university students participated in the EFA phase. The age of the respondents ranged from 19 to 29 (*M* age = 23.62, *SD* = 2.74). More than half (50.8%, *n* = 127) of the participants were female, 31.6% (*n* = 79) were married, and 59.6% (*n* = 149) had a doctorate. About 23.2% (*n* = 58) of them studied dentistry, and 86% (*n* = 215) of them were unemployed. Nearly 96.5% (*n* = 241) of the participants had no experience of MA use. Table 1 provides the participants’ descriptive characteristics in three analyses—EFA, CFA, and test-retest.

### Construct validity

#### Exploratory factor analysis

**Fig. 1:**
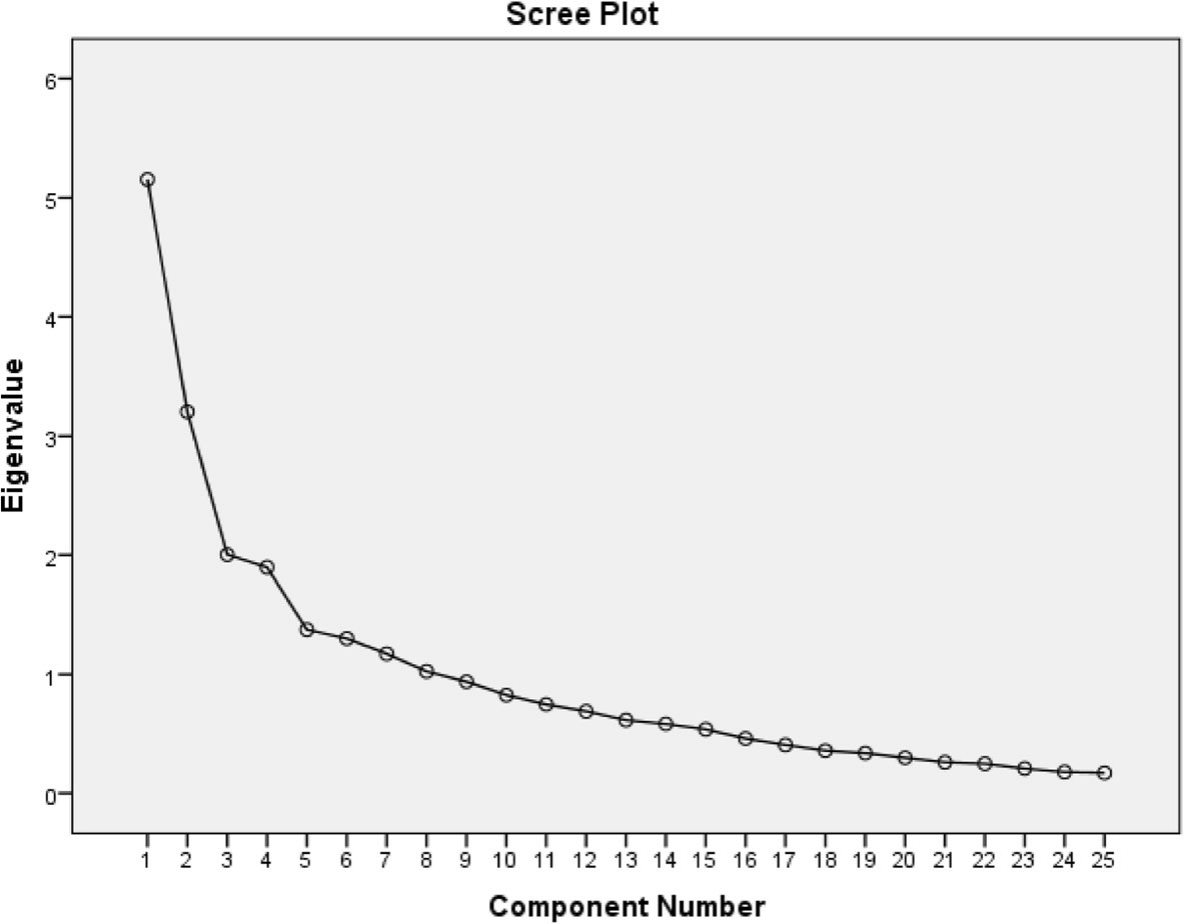
Scree plot for determining the factors

The KMO measure was .733, and Bartlett’s test of sphericity was significant (χ2 = 2096.18, *p* < .001), demonstrating the adequacy of the sample for EFA. At the beginning, for the 25-item scale, eight factors revealed eigenvalues greater than 1, explaining the 68.55% variance, but the scree plot revealed a 4-factor solution (Fig. 1). This 4-factor solution was explored by measuring item performance with deletion of the items in a step-by-step process. Using a varimax rotation, items were kept if they loaded .40 or above on one factor. Four items that did not meet the factor loading criteria were removed. Thereafter, item loadings were again tested and a final factor solution that consisted of a 21-item scale loading on four distinct constructs was obtained. The four factors were different from each other, both statistically and theoretically, and jointly accounted for 55.24% of the observed variance.

As shown in Table 2, four factors were found: factor 1 (self cons) included 7 items (items 1, 2, 3, 4, 5, 6, and 7), factor 2 (coping pros) included 6 items (items 16, 17, 18, 19, 20, and 21), factor 3 (social pros) included 4 items (items 10, 11, 12, and 13), and factor 4 (other cons) included 4 items (items 8, 9, 14, and 15). See Appendix 1 for the items of the METH-DBS .

**Table 2:** Exploratory factory analysis of the METH-DBS (*n* = 250)

Item	Factor 1	Factor 2	Factor 3	Factor 4
6. It damages my judgment, which may threaten myself or others.	**.769**	−.004	.165	−.010
5. It conflicts with my functioning at home and/or at work.	**.754**	.064	.184	−.084
1. It’s illegal, and I will have to worry about getting arrested.	**.729**	.113	.054	.287
2. It could have unpleasant psychological effects (e.g., paranoia, hallucinations, memory loss, and mood disturbances).	**.718**	.125	.092	.251
3. It might be harmful to my body (e.g., brain, liver, heart).	**.704**	.023	.239	−.018
7. It causes me to feel more disobedient or unconventional.	**.692**	.104	−.156	.209
4. It could intensify as a “gateway drug,” leading to other hazardous drug use.	**.586**	.120	.360	.022
19. It will increase and improve my sex.	.034	**.755**	−.059	.102
20. It will help me to centralize and be more creative.	−.045	**.713**	−.210	.074
18. It could make me more relaxed or provide comfortable sleep.	.068	**.700**	.001	−.022
21. It helps me to cope better with disappointment.	.118	**.664**	−.192	.041
16. It will relieve tension, worry, fear, or anxiety.	.128	**.652**	.096	−.259
17. It is something entertaining and breathtaking to do, especially if I’m tired.	.099	**.541**	.189	−.166
13 Using methamphetamine would make others respect me more.	.083	−.055	**.786**	.179
12. Using methamphetamine will make others understand me more positively (e.g., calm, fun, friendly).	.120	−.036	**.752**	.119
10. It will provide chances for social activities (e.g., meeting new friends, grouping, spending time with others).	.208	−.156	**.595**	−.121
11. It’s an escape from truth and daily life.	.182	.023	**.577**	.059
8. It causes family members and/or coworkers to not respect me.	.288	−.081	−.147	**.752**
9. It’s not approved of by the persons who are significant to me.	.346	−.126	−.001	**.738**
15. It causes me to accidentally hurt others due to my daily use.	−.006	−.031	.408	**.659**
14. It causes some persons close to me to become disappointed in me due to my daily use.	−.097	.063	.373	**.550**

*Note* Figures in bold are related to factor loadings equal to or greater than 0.50

#### Confirmatory factor analysis (CFA)

**Fig. 2:**
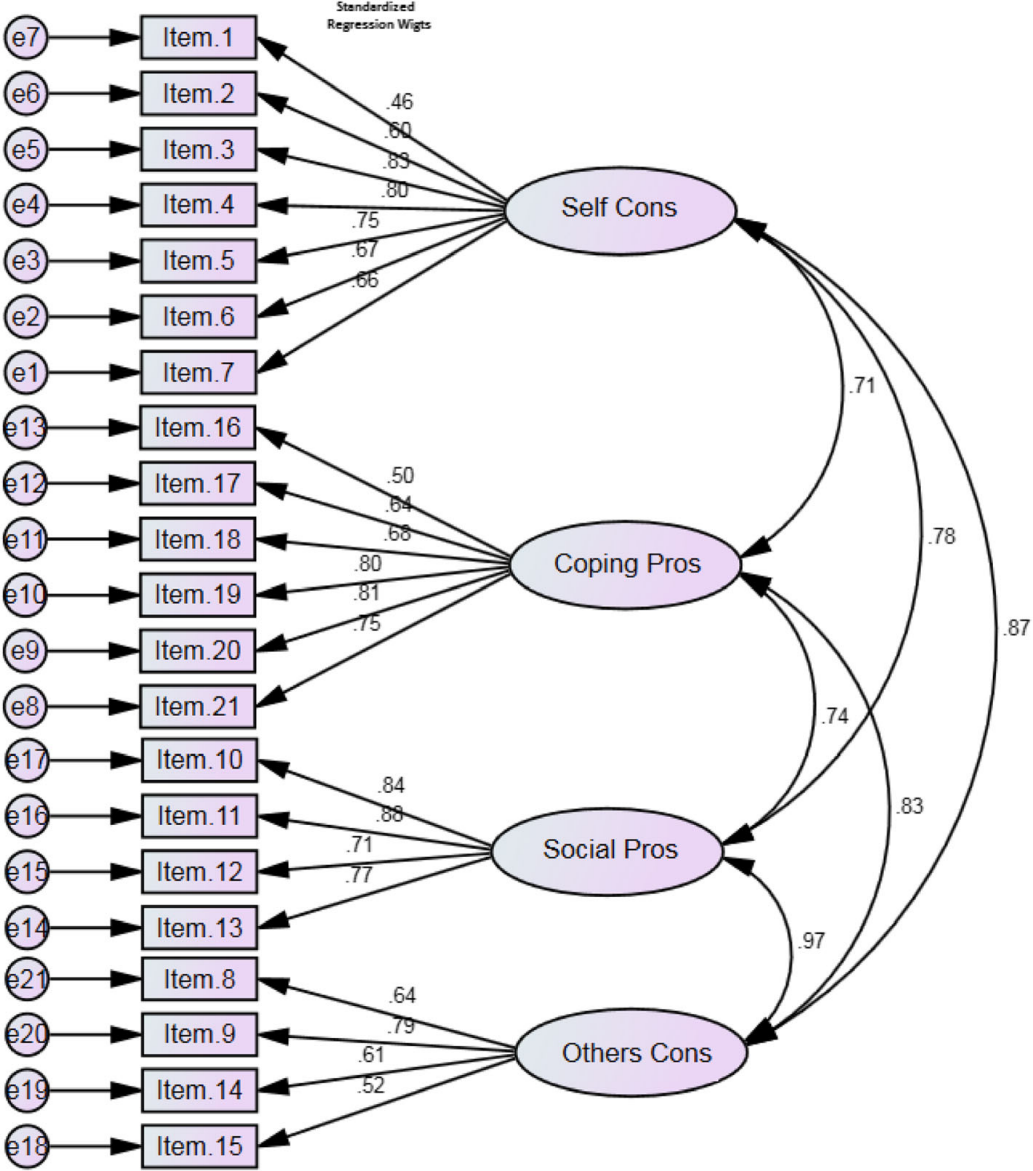
A four-factor model for the questionnaire obtained from confirmatory factory analysis (*n* = 189)

A CFA was conducted on the 21-item scale to test the fitness of the model obtained from the EFA. The best model fit was obtained by applying covariance matrixes and measuring fit indices. As shown in Fig. 2, all fit indices were satisfactory. The relative chi-square (χ2/df) was equal to 3.51 (*p* < .001). The RMSEA of the model was .079 (90% CI = .061–.1), and the SRMR was .05. All comparative indices of the model, including GFI, CFI, NNFI, and NFI, were more than .70 (.80, .81, .79, and .75, respectively).

#### Convergent-divergent and concurrent validity

Table 3 presents the item-convergent validity for the METH-DBS. As can be seen, all coefficients are higher than .60, and most of them are higher than .70. Social pros and self-cons had the highest and the lowest item-convergent validity, respectively. Convergent validity was assessed by the correlation between the four subscales of the METH-DBS and the subscales of the DBI. The correlation between the self and others cons of the METH-DBS and the cons of the DBI was .736 and .690, respectively, which indicated that the convergent validity was very good. Similarly, the correlations between the social and coping pros of the METH-DBS and the social and coping pros of DBI were .576 and .688, respectively, demonstrating an acceptable convergent validity. Based on Table 4, the other correlations were low (≤ .20), indicating an appropriate divergent validity (Table 4).

**Table 3:** Item-scale correlation matrix for the four METH-DBS measures (*n* = 189)

METH-DBS Dimensions	SC	CP	SP	OC
SC (item number)				
Item 1	**.608**	.268	.396	.366
Item 2	**.730**	.438	.481	.480
Item 3	**.812**	.546	.538	.544
Item 4	**.787**	.443	.526	.518
Item 5	**.749**	.420	.459	.473
Item 6	**.724**	.464	.525	.599
Item 7	**.690**	.473	.512	.571
CP (item number)				
Item 16	.331	**.666**	.422	.591
Item 17	.427	**.783**	.405	.548
Item 18	.358	**.762**	.525	.561
Item 19	.479	**.833**	.499	.551
Item 20	.597	**.768**	.528	.540
Item 21	.544	**.717**	.459	.528
SP (item number)				
Item 10	.658	.576	**.813**	.667
Item 11	.600	.573	**.880**	.657
Item 12	.495	.452	**.838**	.592
Item 13	.546	.527	**.865**	.615
OC (item number)				
Item 8	.564	.395	.568	**.680**
Item 9	.631	.531	.719	**.765**
Item 14	.502	.627	.503	**.781**
Item 15	.401	.617	.457	**.743**

*Note. SC* Self Cons, *CP* Coping Pros, *SP* Social Pros, *OC* Others Cons

The bold data reflect higher correlations for the four METH-DBS dimensions

**Table 4:** Correlations between some METH-DBS domains and the DBI

METH-DBS Dimensions	Correlation with DBI Dimensions		
	Cons	Social Pros	Coping Pros
Self Cons	**.736**	.688	.717
Others Cons	**.690**	.535	.569
Social Pros	.427	**.576**	.515
Coping Pros	.453	.484	**.688**

*Note.* The bold data reflect higher correlations between the METH-DBS domains and the three dimensions of the DBI

### Reliability

In order to measure reliability, Cronbach’s alpha was calculated separately for the METH-DBS as a whole and for each dimension of the METH-DBS. Cronbach’s alpha coefficient for the METH-DBS was .933 and ranged from .736 to .871 for its subscales, which is well above the acceptable threshold. Consequently, no items of the questionnaire were deleted in this phase. In addition, test-retest analysis was conducted to test the stability of the instrument. The results indicated satisfactory reliability. The ICC was .957 for the METH-DBS and ranged from .832 to .907 for its subscales, lending support for the stability of the questionnaire. The results are presented in Table 5.

**Table 5:** Measures of internal consistency and stability

Factor	The name of factor	Number of items	Cronbach alpha (*n* = 189)	ICC (*n* = 30)
1	Self Cons	7 items (1–7)	0.849	.907
2	Coping Pros	6 items (16–21)	0.842	.899
3	Social Pros	4 items (10–13)	0.871	.880
4	Others Cons	4 items (8, 9, 14, 15)	0.736	.832
Total		21 items	0.933	.957

## Discussion

In this study, we developed a decisional balance scale to measure the costs and benefits of MA use (METH-DBS) among young adults. This is the first study to provide a measure for evaluating the items associated with the costs and benefits of MA use in Iranian young adults. The content of the scale items was first developed based on a qualitative study to ensure that this scale covered all theoretical concepts related to the costs and benefits of MA use. After EFA, a four-domain scale emerged. A CFA showed that the fit of the data was acceptable. As such, the final METH-DBS scale contained 21 items, with 7 items representing self cons, 6 items representing coping pros, 7 items representing social pros, and 4 items representing other cons of MA use. These results are consistent with studies that developed DB constructs for other health/risk behaviors [22]. Reliability analyses indicated strong internal consistency (α > .90). As such, we believe the METH-DBS scale represents a new scale for understanding the motivations to use MA.

Items included in the self-cons subscales reflect negative consequences of MA use on users that might encourage/discourage persons to make decisions for changing behavior. Items included in the other cons subscales reflect the negative consequences of MA use for significant others. The cons subscale about MA use could help practitioners because it includes factors that facilitate preventive behaviors about MA use, including issues related to clients’ personal and social concerns.

Items included in the social pros subscale involve the socially positive aspects of MA use from the users’ perspective. Additionally, the coping pros subscale includes items that cover a wide range of reasons for using MA to control people’s levels of tension and worry, usually for the purpose of improving everyday activities. Pros have a great impact on performing risky behaviors and are associated with increasing MA use, so pros are of great importance in the issue of behavioral change. It is important to know that people who had more positive expectations about ceasing use of MA or other risky behaviors felt more efficacious about performing behaviors such as enhancing chances for social activities; being calm; relieving tension, worry, and fear [18, 22, 47]. Changing risky behaviors, especially drug addiction behaviors, requires long-term investments. Therefore, it is unlikely for this group to agree to such behaviors out of habit without any conscious decision to do so.

Several factors reinforced the pros of MA use, including the increased rate of MA use, more problems that led people to use MA for stress relief, and intentions to use in the future, as well as stronger positive beliefs and attitudes for legalizing MA. By showing the negative effects and cons of MA use, use might decrease in youngsters. Greater endorsement of cons was associated with a decreased rate of use and fewer intentions to use drugs, which is consistent with other studies displaying an adverse relation between use rate and perceived risk [48]. Participants who recognized cons held more beliefs about negative consequences and were unlikely to favor legalizing MA. However, cons were incoherently associated with MA-related problems. Similarly, in a study by Noar et al. (2003) about alcohol DB, it was mentioned that even clients experiencing few problems recognize that there are cons to consuming MA [49]. The possibility for social interest bias was perceived for pros in one sample, but there was no relationship with cons. Therefore, participants who tried to present themselves in a socially acceptable light were unwilling to admire the pros of MA use, perhaps because it is an illegal drug. The potential for social desirability bias should be considered in future investigations using this scale.

The association between MA pros and cons across stages of change provides cross-sectional support for predictions from the TTM of change. Specifically, the “crossover effect” appears to take place on the verge of the preparation and action stages, which is late in the phases based on some behaviors like safe sex, but it estimates the point at which crossover happens for other behaviors such as regular physical activity [22].

Generally, our findings indicated satisfactory psychometric properties for the METH-DBS. The CVI and the CVR showed that the content validity was acceptable. Additionally, the findings support the construct validity of the pros (coping and social) and cons (self and other) dimensions, as the results of the EFA and CFA indicated a good structure for our new instrument. EFA showed that the four-factor structure of the scale accounted for 55.24% of the total observed variance. The cautious selection of items for the instrument may be the reason why we have attained such satisfactory results. In addition, the CFA also displayed good fit indices for the existing model, and the convergent validity of the dimensions of the scale was good with a total score between .61 and .88. Furthermore, the pros (coping and social) and cons (self and other) in the final instrument reveal several of the content domains mentioned by Janis and Mann (1977) [16], counting personal gains/losses from MA use (e.g., “It would relieve tension, worry, fear, or anxiety”), gains/losses for others (e.g., “It causes me to accidentally hurt others due to my daily use”), and items about approval and disapproval from important others (e.g., “It’s not approved of by persons who are significant to me”). Based on item valence, many items show agreement or disagreement. However, there was no retained item that directly referenced self-approval or disapproval.

The internal consistency of the final scale, as measured by Cronbach’s alpha coefficient, was found to be .933, which reflected acceptable reliability. Furthermore, the ICC score showed suitable stability for the scale as it was tested by 30 college students with a 2-week interval (.957). As such, we believe that this newly generated scale may be especially useful for health care teams to understand and design approaches that are practical and targeted to specific situations. The inclusion of four domains in this scale further allows experts to know how domains in which a person has needs can be boosted.

### Limitations

Although the results of the present work confirmed several benefits, some limitations must be addressed, as with any other study. First, relating to the participants, we only interviewed medical university students at a single medical university in Zanjan, Iran, which possibly limited the external validity of this scale. The young adults of this sample are at high risk for MA use, and both genders and several ethnicities are represented in the sample. Although students vary both across and within countries as much as the general public [50], (medical) college students are however not representative of the general population. They are younger, higher educated, keep different hours, have different habits and may have different values. Given that some medical college students’ courses are related to drug related topics, it seems that these students have more information about the addiction and types of drugs. Consequently, data gleaned from our sample cannot be generalized to the viewpoint of university students studying in other universities of Iran nor to the general population. Thus, it might be interesting for future research to study the reliability and validity of the METH-DBS in a sample of young adults, with different backgrounds, and from various regions. Second, we used two different samples for our EFA and CFA. Although the same method was performed to gather data from the participants, some background information of the students was not the same, particularly the experience of MA use, employment status, and field of study. This might have influenced the results of the present study. Fourth, although MA users were overrepresented compared to the population prevalence, the inclusion of abstainers may have led to the retaining of some items that would not have been used by MA users and may be linked to the large number of cons in the final instrument. Last, generalizability to non-university-attending adults cannot be expected; further investigation with other populations must be performed before the instrument is applied that would help to determine the usefulness of the METH-DBS. Duplicating the factor structure with different samples (e.g., those with MA dependence) could shed light on its generalizability. Likewise, validating the instrument with other participants would support its usefulness beyond young adults who are attending a university.

In summary, one of the objectives for the century is preventing and controlling high-risk behavior such as MA use [51]. To do so, we developed the METH-DBS, which was demonstrated to have satisfactory psychometric properties. The METH-DBS measures pros and cons of MA use that help to promote individuals’ health.

## Conclusion

In general, the present findings show that the METH-DBS is a valid and reliable instrument to assess the pros and cons of MA use. Further studies with participants from different backgrounds are suggested to find stronger psychometric properties for the scale.

## Appendix 1

**Self Cons**

1. It’s illegal, and I will have to worry about getting arrested.

2. It could have unpleasant psychological effects (e.g., paranoia, hallucinations, memory loss, mood disturbances).

3. It might be harmful to my body (e.g., brain, liver, heart).

4. It could intensify as a “gateway drug,” leading to other hazardous drug use.

5. It conflicts with my functioning at home and/or at work.

6. It damages my judgment, which may threaten me or others.

7. It makes me feel out of control.

**Other Cons**

8. It causes family members and/or coworkers to not respect me.

9. It’s not approved of by the persons who are significant to me.

14. It causes some persons close to me to become disappointed in me due to my daily use.

15. It causes me to accidentally hurt others due to my daily use.

**Social Pros**

10. It will provide chances for social activities (e.g., meeting new friends, grouping, spending time with others).

11. It’s an escape from truth and daily life.

12. Using methamphetamine will make others understand me more positively (e.g., calm, fun, friendly).

13. Using methamphetamine would make others respect me more.

**Coping Pros**

16. It will relieve tension, worry, fear, or anxiety.

17. It is something entertaining and breathtaking to do, especially if I’m tired.

18. It could make me more relaxed or provide comfortable sleep.

19. It will increase and improve my sex life.

20. It will help me to center myself and be more creative.

21. It helps me to cope better with disappointment.

## Abbreviations

CFA: Confirmatory factor analysis
CFI: Comparative Fit Index
Cons: Negative thoughts
CVI: Content validity index
CVR: Content validity ratio
DBI: Decisional Balance Inventory
EFA: Exploratory factor analysis
GFI: Goodness of Fit Index
ICC: Intraclass correlation coefficient
METH-DBS: Methamphetamine Decisional Balance Scale
NFI: Normed Fit Index
NNFI: Non-Normed Fit Index
Pros: Positive thoughts
RMSEA: Root Mean Square Error of Approximation
SRMR: Standardized Root Mean Square Residual
TTM: Transtheoretical model

## References

[1] World Health Organization. Drug use and road safety: a policy brief. Geneva, Switzerland, 2016. http://www.who.int/substance_abuse/publications/drugs/en/. Accessed 19 Dec 2016.

[2] Chauhan H, Killinger BA, Miller CV, Moszczynska A (2014). Single and binge methamphetamine administrations have different effects on the levels of dopamine D2 autoreceptor and dopamine transporter in rat striatum. Int J Mol Sci.

[3] United Nations Office on Drugs and Crime (UNODC) (2015). World Drug Report.

[4] Marshall JF, O'Dell SJ (2012). Methamphetamine influences on brain and behavior: unsafe at any speed?. Trends Neurosci.

[5] Brackins T, Brahm NC, Kissack JC (2011). Treatments for methamphetamine abuse: a literature review for the clinician. J Pharm Pract.

[6] Sobieraj JC, Kim A, Fannon MJ, Mandyam CD (2016). Chronic wheel running-induced reduction of extinction and reinstatement of methamphetamine seeking in methamphetamine dependent rats is associated with reduced number of periaqueductal gray dopamine neurons. Brain Struct Funct.

[7] Norberg Melissa M., Kezelman Sarah, Lim-Howe Nicholas (2013). Primary Prevention of Cannabis Use: A Systematic Review of Randomized Controlled Trials. PLoS ONE.

[8] Glasner-Edwards S, Mooney LJ, Marinelli-Casey P, Hillhouse M, Ang A, Rawson RA (2010). Methamphetamine treatment project corporate authors. Psychopathology in methamphetamine-dependent adults 3 years after treatment. Drug Alcohol Rev..

[9] McKetin R, Lubman DI, Najman JM, Dawe S, Butterworth P, Baker AL (2014). Does methamphetamine use increase violent behaviour? Evidence from a prospective longitudinal study. Addiction.

[10] Braun V, Clarke V (2006). Using thematic analysis in psychology. Qual Res Psychol.

[11] Karrari P, Mehrpour O, Balali-Mood M (2012). Iranian crystal: a misunderstanding of the crystal-meth. J Res Med Sci.

[12] Shariatirad S, Maarefvand M, Ekhtiari H (2013). Emergence of a methamphetamine crisis in Iran. Drug Alcohol Rev.

[13] Ishaak F, de Vries NK, van der Wolf K (2015). Design of study without drugs--a Surinamese school-based drug-prevention program for adolescents. BMC Public Health.

[14] Faggiano F, Minozzi S, Versino E, Buscemi D (2014). Universal school-based prevention for illicit drug use. Cochrane Database Syst Rev.

[15] Porath-Waller AJ, Beasley E, Beirness DJ (2010). A meta-analytic review of school-based prevention for cannabis use. Health Educ Behav.

[16] Janis IL, Mann L (1977). Decision making: a psychological analysis of conflict, choice, and commitment.

[17] Apodaca TR, Longabaugh R (2009). Mechanisms of change in motivational interviewing: a review and preliminary evaluation of the evidence. Addiction.

[18] Prochaska JO, Velicer WF (1997). The transtheoretical model of health behavior change. Am J Health Promot.

[19] Prochaska JO (1996). A stage paradigm for integrating clinical and public health approaches to smoking cessation. Addict Behav.

[20] Girma E, Assefa T, Deribew A (2010). Cigarette smokers' intention to quit smoking in Dire Dawa town Ethiopia: an assessment using the Transtheoretical model. BMC Public Health.

[21] Khazaee-Pool M, Pashaei T, Ponnet K, Jafari F, Alizadeh R (2017). Correction to: decisional balance inventory (DBI) adolescent form for smoking: psychometric properties of the Persian version. BMC Public Health.

[22] Velicer WF, DiClemente CC, Prochaska JO, Brandenburg N (1985). Decisional balance measure for assessing and predicting smoking status. J Pers Soc Psychol.

[23] Lea T, Kolstee J, Lambert S, Ness R, Hannan S, Holt M (2017). Methamphetamine treatment outcomes among gay men attending a LGBTI-specific treatment service in Sydney, Australia. PLoS One.

[24] Braun V, Clarke V (2014). What can “thematic analysis” offer health and wellbeing researchers?. Int J Qual Stud Health Well-being.

[25] Khazaee-Pool M, Majlessi F, Montazeri A, Pashaei T, Gholami A, Ponnet K (2016). Development and psychometric testing of a new instrument to measure factors influencing women's breast cancer prevention behaviors (ASSISTS). BMC Womens Health.

[26] Waltz CF, Bausell BR. Nursing research: design statistics and computer analysis. First ed. Davis FA; 1981.

[27] Waltz CF, Strickland OL, Lenz ER. Measurement in nursing and health research. 4rd ed. New York: Springer; 2005.

[28] Polit DF, Beck CT (2006). The content validity index: are you sure you know what's being reported? Critique and recommendations. Res Nurs Health.

[29] Lawshe CH (1975). A quantitative approach to content validity. Pers Psychol.

[30] Khazaee-Pool M, Pashaei T, Ponnet K, Jafari F, Alizadeh R (2017). Decisional balance inventory (DBI) adolescent form for smoking: psychometric properties of the Persian version. BMC Public Health.

[31] Khazaee-Pool M, Arefi Z, Roshani D, Pashaei T (2017). Development and psychometric properties of a questionnaire to measure drug users' attitudes toward methadone maintenance treatment (DUAMMT) in Iran. BMC Public Health.

[32] Lacasse Y, Godbout C, Sériès F (2002). Health-related quality of life in obstructive sleep apnoea. Eur Respir J.

[33] Pallonen UE, Prochaska JO, Velicer WF, Prokhorov AV, Smith NF (1998). Stages of acquisition and cessation for adolescent smoking: an empirical integration. Addict Behav.

[34] Grant JS, Davis LL (1997). Selection and use of content experts for instrument development. Res Nurs Health.

[35] McCoach D. Betsy, Gable Robert K., Madura John P. (2013). Instrument Development in the Affective Domain.

[36] Ferguson E, Cox T (1993). Exploratory factor analysis: a users’ guide. Int J Sel Assess.

[37] Norris M, Lecavalier L (2010). Evaluating the use of exploratory factor analysis in developmental disability psychological research. J Autism Dev Disord.

[38] Harrington D (2009). Confirmatory factor analysis.

[39] Kline RB. Principles and practice of structural equation modeling. 3rd ed. New York: Guilford publications; 1998.

[40] Schumacker RE, Lomax RG. A beginner's guide to structural equation modeling. 3^rd^ ed. New York, NY: Routledge; 2010.

[41] Nunnally J, Bernstein I (1994). Psychometric theory.

[42] Fayers PM, Machin D. Quality of life: the assessment, analysis and interpretation of patient-reported outcomes. 2nd ed: Wiley; 2013.

[43] Cronbach LJ (1951). Coefficient alpha and the internal structure of tests. Psychometrika.

[44] Munro BH. Statistical methods for health care research. 5th ed. Philadelphia: Wolters Kluwer health/Lippincott Williams & Wilkins Publisher; 1986.

[45] Corp I. IBM SPSS statistics for windows, version 22.0. Armonk, NY: IBM Corp. 2013.

[46] Arbuckle J. SPSS Amos (version 22.0) [computer program]. Chicago, IL: SPSS. 2006.

[47] Berry T, Naylor PJ, Wharf-Higgins J (2005). Stages of change in adolescents: an examination of self-efficacy, decisional balance, and reasons for relapse. J Adolesc Health.

[48] Kelly BC, Liu T, Yang XY, Zhang G, Hao W, Wang J (2014). Perceived risk of methamphetamine among Chinese methamphetamine users. Int J Drug Policy.

[49] Noar SM, Laforge RG, Maddock JE, Wood MD (2003). Rethinking positive and negative aspects of alcohol use: suggestions from a comparison of alcohol expectancies and decisional balance. J Stud Alcohol.

[50] Hanel PH, Vione KC (2016). Do student samples provide an accurate estimate of the general public?. PLoS One.

[51] Hendershot CS, Witkiewitz K, George WH, Marlatt GA (2011). Relapse prevention for addictive behaviors. Subst Abuse Treat Prev Policy.

